# Acyloxyacyl hydrolase regulates microglia-mediated pelvic pain

**DOI:** 10.1371/journal.pone.0269140

**Published:** 2022-08-18

**Authors:** Afrida Rahman-Enyart, Ryan E. Yaggie, Justin L. Bollinger, Constadina Arvanitis, Deborah R. Winter, Anthony J. Schaeffer, David J. Klumpp

**Affiliations:** 1 Department of Urology, Feinberg School of Medicine, Northwestern University, Chicago, Illinois, United States of America; 2 Currently Proteintech Group Incorporated, Rosemont, Illinois, United States of America; 3 Department of Pharmacology & Systems Physiology, College of Medicine University of Cincinnati, Cincinnati, Ohio, United States of America; 4 Department of Cell and Developmental Biology, Feinberg School of Medicine, Northwestern University, Chicago, Illinois, United States of America; 5 Center for Advanced Microscopy & Nikon Imaging Center, Feinberg School of Medicine, Northwestern University, Chicago, Illinois, United States of America; 6 Division of Rheumatology, Feinberg School of Medicine, Northwestern University, Chicago, Illinois, United States of America; 7 Department of Microbiology-Immunology, Feinberg School of Medicine, Northwestern University, Chicago, Illinois, United States of America; University of Arizona College of Medicine, UNITED STATES

## Abstract

Chronic pelvic pain conditions such as interstitial cystitis/bladder pain syndrome (IC/BPS) remain clinical and mechanistic enigmas. Microglia are resident immune cells of the central nervous system (CNS) that respond to changes in the gut microbiome, and studies have linked microglial activation to acute and chronic pain in a variety of models, including pelvic pain. We have previously reported that mice deficient for the lipase acyloxyacyl hydrolase (AOAH) develop pelvic allodynia and exhibit symptoms, comorbidities, and gut dysbiosis mimicking IC/BPS. Here, we assessed the role of AOAH in microglial activation and pelvic pain. RNAseq analyses using the ARCHS4 database and confocal microscopy revealed that AOAH is highly expressed in wild type microglia but at low levels in astrocytes, suggesting a functional role for AOAH in microglia. Pharmacologic ablation of CNS microglia with PLX5622 resulted in decreased pelvic allodynia in AOAH-deficient mice and resurgence of pelvic pain upon drug washout. Skeletal analyses revealed that AOAH-deficient mice have an activated microglia morphology in the medial prefrontal cortex and paraventricular nucleus, brain regions associated with pain modulation. Because microglia express Toll-like receptors and respond to microbial components, we also examine the potential role of dysbiosis in microglial activation. Consistent with our hypothesis of microglia activation by leakage of gut microbes, we observed increased serum endotoxins in AOAH-deficient mice and increased activation of cultured BV2 microglial cells by stool of AOAH-deficient mice. Together, these findings demonstrate a role for AOAH in microglial modulation of pelvic pain and thus identify a novel therapeutic target for IC/BPS.

## Introduction

Interstitial cystitis/ bladder pain syndrome (IC/BPS) is a debilitating condition characterized by chronic pelvic pain and associated with lower urinary tract symptoms and gut dysbiosis [1–4]. Treating IC/BPS is challenging due to unknown etiology and a lack of biomarkers, making advances in understanding IC/BPS a necessity [5]. Towards this goal, we previously conducted a genetic screen in a mouse model of neurogenic cystitis and identified a polymorphism associated with pelvic pain severity near the gene encoding acyloxyacyl hydrolase, *Aoah* [6].

AOAH is a highly conserved lipase responsible for hydrolyzing the secondary fatty acyl chains of gram-negative bacterial lipopolysaccharides (LPS), resulting in LPS detoxification and attenuation of host inflammation [7–11]. We have previously reported that mice deficient for AOAH exhibit pelvic allodynia and increased responses in induced pelvic pain models [6]. Furthermore, we have shown that AOAH deficiency enhances arachidonic acid-dependent expression of corticotropin releasing factor (CRF) in the paraventricular nucleus (PVN) of the hypothalamus [12], a pertinent brain region for CRF-dependent pain modulation [13, 14]. Finally, AOAH-deficient mice mimic other facets of IC/BPS, including an anxious/depressive phenotype, altered voiding, and dysbiosis of the gut microbiome [2, 12, 15–19].

Gut microbiota and associated metabolites are key regulators of the immune response, and play an important role in the development and maturation of microglia [20–22]. Critical regulators of brain development and homeostasis, microglia are intrinsic immune cells of the central nervous system (CNS) that are highly similar to macrophages—indeed are often considered CNS macrophages—including acting as phagocytes, secreting cytokines in response to Toll-like receptor activation, and displaying multiple polarized phenotypes [23, 24]. Rodent models have revealed a critical role for microglia in modulating acute and chronic pain ([25, 26] for review), including acute cystitis and chronic prostatitis pelvic pain models [27–30]. Microglia exhibit a unique morphological structure, consisting of a largely stable cell body with an array of highly dynamic surveillant processes. In response to environmental cues, microglia can shift their morphology from this surveillant mode to a bushy or ameboid phenotype. This shift in morphology is associated with increased cytokine release, microglial proliferation, migration, and phagocytosis [31].

The gut microbiome has previously been shown to regulate microglial function [21, 22, 32], and microbiome-microglial interactions are implicated in the development and maintenance of chronic pain [20, 33–36]. Several mechanisms of how gut microbes may interact with microglia and regulate chronic pain have been proposed, including the leakage of bacterial products across an impaired gut barrier to the brain [20]. Previous studies have shown that LPS can directly activate microglial cells [37–39]. Since AOAH-deficient mice exhibit compromised gut epithelia and gut dysbiosis [19], we hypothesized that AOAH deficiency would result in microglial activation. Indeed, we observed that AOAH-deficient mice exhibited activated microglia in brain regions important for pain modulation. Furthermore, we observed that microglia regulated pelvic pain in AOAH-deficient mice, as microglial ablation partially alleviated the pelvic pain phenotype. These findings suggest that microglial activation can regulate pelvic pain and provide insights for future development of therapeutics for pelvic pain disorders such as IC/BPS.

## Materials and methods

### Animals

All animal experiments were performed in accordance to protocols approved by the Institutional Animal Care and Use Committee of Northwestern University. Eight to ten-week-old female wild type (WT) C57BL/6 mice were purchased from The Jackson Laboratory (Bar Harbor, ME). *Aoah*^—/—^mice (B6.129S6-*Aoah*^*tm1Rsm*^/J) were a generous gift from Dr. Robert Munford of NIAID and maintained as previously described [12]. Mice were housed in facilities of the Center for Comparative Medicine of Northwestern University and maintained on a 12h:12h light:dark cycle.

### Patients and sample collection

Studies were conducted under Protocol STU00055668 and approved by the Institutional Review Board of Northwestern University and carried out in accordance with the approved procedures. Female participants (IC patients and healthy controls) were recruited into the Urology Clinic of Northwestern University Feinberg School of Medicine as previously described [2]. Patients were enrolled using standard Pelvic Pain inclusion and exclusion criteria. Pelvic Pain inclusion criteria are: Must be at least 18 years old; Participant reports an unpleasant sensation of pain, pressure or discomfort, perceived to be related to the bladder and/or pelvic region, associated with lower urinary tract symptoms (LUTS); Symptoms have been present for the majority of the time during any 3 months in the previous 6 months; and Symptoms have been present for the majority of the time during the most recent 3 months. Patients were excluded if: Under the age of 18; Participant has an on-going symptomatic urethral stricture; Participant has an on-going neurological disease or disorder affecting the bladder or bowel fistula; Participant has a history of cystitis caused by tuberculosis, radiation therapy or Cytoxan/cyclophosphamide therapy; Participant has augmentation cystoplasty or cystectomy; Participant has an active autoimmune or infectious disorder (such as Crohn’s Disease or Ulcerative Colitis, Lupus, Rheumatoid Arthritis, Multiple Sclerosis, or HIV); Participant has a history of cancer (with the exception of skin cancer); Participant has any psychiatric or medical comorbidities that would interfere with study participation (e.g. dementia, psychosis, upcoming major surgery, lupus, active heart failure, diabetes etc); Participant currently has a urinary tract infection (UTI) and/or has had a positive urine culture in the past 6 weeks; and Participant is currently taking antibiotics or has in the last 3 months.

Demographic data and symptom scores were assessed using a female-specific genitourinary pain index (GUPI) questionnaire collected at the time of enrollment (S1 Table for information on patients included in the current study) [40]. Participant stool samples were collected according to the approved protocols of the HMP Manual of Procedures and previously described in Braundmeier-Fleming et al. [2]. Briefly, participants were provided an at-home stool collection kit and instructed to collect a sample followed by overnight freezing. Samples were then shipped on wet ice to the laboratory within 48 hr after collection. Stool samples were then aliquoted, placed into RNAlater (MoBio; Jefferson City, MO), and stored at −80°C for later use. Samples for the current study were selected by total GUPI score. A score of 0 was required for healthy controls (n = 2) and a score above 30 was required for IC patients (n = 3).

### ARCHS4 RNAseq database

AOAH cell and tissue expression was obtained from the ARCHS4 database of 187,964 human and mouse samples as previously described [41]. Utilizing their “Enter Gene Symbol” search function, we queried the keyword “AOAH” to obtain gene counts from HiSeq 2000, HiSeq 2500 and NextSeq 500 platforms for human and mouse experiments from NCBI Gene Expression Omnibus (GEO) and Sequence Read Archive (SRA). The resulting map of AOAH relative expression in 72 cells or tissues was exported directly from the database.

### Immunohistochemistry

Coronal brain sections for immunohistochemistry were prepared from WT and *Aoah*^—/—^mice. Mice were first anesthetized with isoflurane and then transcardially perfused for 2 min with phosphate buffered saline (PBS) followed by 4% paraformaldehyde for 10 min. Brains were further fixed in 4% paraformaldehyde overnight and equilibrated successively in 15 and 30% sucrose in PBS for cryoprotection. Tissues were then frozen in dry ice and embedded in Tissue Plus optimum cutting temperature medium (Fisher HealthCare, Houston, TX). Cryostat sections of 40 μm were collected and placed into 24-well plates with antifreeze (30% ethyleneglycol, 30% glycerol, 10% 2 X PO_4_ buffer (0.244M) in dH_2_0) and stored at -20°C. Prior to staining, free floating sections were washed 2 x 5 min in PBS and incubated for 1 hr at room temperature with blocking solution (1% bovine serum albumin (Gemini Bio Products, West Sacramento, CA) in PBS). Sections were then incubated overnight at 4°C with the following antibody dilutions in blocking solution: goat polyclonal anti-Iba1 (1:500; Abcam, Cambridge, United Kingdom, ab107159), rabbit polyclonal anti-P2RY12 (1:1000; AnaSpec, Fremont, CA, AS-55043A), mouse monoclonal anti-GFAP (1:500; Abcam, ab10062), mouse monoclonal anti-AOAH (1:100; Santa Cruz Biotechnology, Dallas, TX, sc-163692), rabbit polyclonal anti-AOAH (1:100; Santa Cruz Biotechnology, sc-135110), and mouse monoclonal anti-CD68 (1:500; Bio-Rad, Hercules, CA, MCA1957GA). Brain sections were then washed 4 x 5 min with PBS followed by overnight incubation at 4°C with the following secondary antibody dilutions in blocking solution: Alexa Fluor 488-goat anti-rabbit (1:1000; Thermo Fisher Scientific, A-11008), Alexa Fluor 488-goat anti-mouse (1:1000; Thermo Fisher Scientific, A-11029), Alexa Fluor 594-donkey anti-rabbit (1:1000; Thermo Fisher Scientific, A-21207), Alexa Fluor 594-donkey anti-mouse (1:1000; Thermo Fisher Scientific, A-21203), and Alexa Fluor 594-donkey anti-goat (1:1000; Thermo Fisher Scientific, A-11058). Secondary antibodies were washed off with PBS (2 x 5 min), incubated for 10 min in PBS/10 μg/mL 4’-6-diamidino-2-phenylindole (DAPI, Thermo Fisher Scientific) to stain nuclei, and further washed in PBS (3 x 5 min). Sections were then mounted onto gel-coated slides, dried for 30 min, and slides were mounted with 60 × 22 mm cover glass (VWR, Radnor, PA) using Clear-Mount Mounting Solution (Thermo Fisher Scientific) prior to imaging.

Z-stacks (3 μm steps) were imaged using 20X and 40X objectives on a Leica DM IRE2 microscope (Leica Biosystems, Wetzlar, Germany) and Volocity 5.0 software (Perkin Elmer, Waltham, MA). Confocal z-stacks were imaged using a 60X objective on a Nikon AX-R Confocal Microscope System (Nikon, Minato City, Tokyo, Japan) and NIS-Elements imaging software (Nikon).

Z-stacks were combined and immunofluorescence was quantified using ImageJ 1.52q software (National Institute of Health and the Laboratory for Optical and Computational Instrumentation) as previously reported in Jensen, 2013 [42]. Briefly, combined z-stack fluorescence images, containing only the channel of interest, were opened in ImageJ. Using the “Threshold” plugin, images were set to “Dark background” for fluorescence and threshold was set. Next, measurements were set by going to “Analyze-Set Measurements” and selecting “Integrated Density.” Integrated pixel density was then measured by selecting “Analyze-Measure.” Data was reported as fluorescence intensity as measured by integrated pixel density.

### PLX5622 treatment

For microglial elimination, colony-stimulating factor-1 receptor (CSF1R) inhibitor PLX5622 was purchased from MedChem Express (Monmouth Junction, NJ, HY-114153) [43]. The drug was prepared following the manufacturer’s instruction by dissolving the drug in 10% DMSO and 90% corn oil. AOAH-deficient mice were gavaged with 90 mg/kg of drug solution for 5 d, allowing for the depletion of majority of CNS microglia as previously reported [43]. Wild type mice were gavaged with 10% DMSO and 90% corn oil only as a control. After 5 d of treatment, we ceased drug administration and allowed microglia to repopulate the CNS.

### Pelvic allodynia

Pelvic allodynia was measured using a modified protocol for traditional allodynia as first reported by Laird et al. [44] and successfully implemented in studies previously done in our laboratory [6, 19, 45, 46]. Briefly, pelvic allodynia was quantified in wild type and *Aoah*^—/—^mice by measuring responses to von Frey filament stimulation of the pelvic region. Mice were singly placed in a test chamber and allowed to acclimate for 5 min. Five von Frey filaments (lowest-to-highest force) were applied 10 times each to the pelvic region and responses were recorded, taking care that each successive stimulis applied at a pelvic site distinct from the prior stimulus. An animal was scored as responsive to the stimulus if it jumped, lifted and shook the hind paws, or excessively licked the pelvic region. Responses for each filament were calculated using the following equation:

$$\%\text{response}=\left(\left(\#\;\text{of}\;\text{evoked}\;\text{responses}\right)/\left(5\;\text{filaments}\;\text{x}\;10\;\text{stimuli}/\text{filament}\right)\right)*100\%$$

### Skeletal analyses and microglial area

Morphological characteristics of microglia were quantified by skeletal analysis as detailed in Young & Morrison [47]. Coronal brain sections labeled for the microglial marker P2RY12 were analyzed in WT and AOAH-deficient mice (n = 15 fields from three different mice for PFC and n = 7 fields from three different mice for PVN). 1344 x 1024-pixel photomicrographs/fields were randomly taken with a 20X objective using a Leica DM IRE2 microscope and Volocity 5.0 software. Images were processed to a binary image and skeletonized using Image J 1.52q software (National Institute of Health and the Laboratory for Optical and Computational Instrumentation), as previously described in detail by Young & Morrison [47]. All microglia were analyzed within each photomicrograph and images were not cropped. Skeletonized images were then run through the AnalyzeSkeleton (2D/3D) plugin to generate data reporting total number of branches and endpoints as well as average and longest process lengths. Outliers that were considered noise were removed prior to data analyses. Outliers were considered to be data containing 2 endpoints with a maximum branch length of less than 1.0 μm or average branch length of less than 1.0 μm. Data was then divided by total number of microglia/image (quantified using the Image J Cell Counter tool) for an average measurement/cell.

Microglial area was measured by first setting the image threshold of photomicrographs labeling P2RY12-positive microglia (described in “Immunohistochemistry”). Using the “Analyze-Set Measurements” feature, “Area” was selected prior to “Analyze-Measure.” The area of the thresholded material was quantified by the software as area in pixels (pixels^2^). Pixel area was then converted to μm^2^ by using the conversion factor of 3.4 pixels/μm. Data was then divided by total number of microglia/image (quantified using the Image J Cell Counter tool) for the mean area/cell.

### Cell culture

The immortalized murine microglial cell line, BV2 were obtained from Elabscience (Wuhan, China, EP-CL-0493). BV2 cells were grown in Minimum Essential Medium (MEM; Thermo Fisher Scientific) containing 10% fetal calf serum (FCS) and 1% penicillin/streptomycin.

### Serum preparation and LAL assay

Serum endotoxin concentration was measured using the Pierce *Limulus* Amebocyte Lysate (LAL) Chromogenic Endotoxin Quantitation Kit (Thermo Fisher Scientific, 88282). Blood samples were collected from WT and AOAH-deficient mice via incision of the tail vain. Samples were allowed to clot for 15 min at room temperature followed by centrifuging at 2,000 x g for 10 min at 4°C. Serum supernatant was then diluted 1:50 with endotoxin-free H_2_O and the assay was performed following the manufacturer’s instructions.

### Western blotting

To prepare lysates for Western blotting, BV2 cells were plated at 500,000 cells/well for 48 hrs. Cells were then activated with lipopolysaccharides (LPS) from *Escherichia coli* 055:B5 (1μg/mL; Millipore Sigma, Burlington, MA), heat-killed stool slurry (65°C for 15 min) prepared from fecal pellets from WT or AOAH-deficient mice homogenized in PBS (1 mg/mL), or heat-killed stool slurry (65°C for 15 min) from healthy or IC/BPS patients homogenized in PBS (1 mg/mL) for 0, 0.5, 1, 2, 6, or 24 hrs. Cells were then lysed for 30 min using RIPA buffer consisting of 50 mM Tris HCl (pH 8.0), 150 mM NaCl, 1 mM EDTA, 1% NP-40, 0.5% sodium deoxycholate, 0.1% SDS, 1% Triton, and protease inhibitor cocktail (Millipore Sigma). After centrifugation and protein determination, cell lysates or cell media were subjected to SDS-PAGE using 4–20% gradient Tris-glycine gels (Bio-Rad) followed by transfer to Immobilon P membranes (EMD Millipore). Following transfer, the membranes were incubated for 30 min in blocking buffer consisting of 10 mM Tris-HCl (pH 8.0), 150 mM NaCl, 0.01% Tween-20 (TBST), and 3% non-fat dry milk (Cell Signaling, Danvers, MA). Membranes were then incubated overnight at 4°C with the following primary antibodies diluted in blocking buffer: rabbit polyclonal anti-CD11b (1:2000; Novus Biologicals, Littleton, CO, NB110-89474), rabbit polyclonal anti-TNF⍺ (1:2000; Proteintech, Rosemont, IL, 17590-1-AP), rabbit polyclonal anti-CD68 (1:2000; Abcam, ab125212), and mouse monoclonal anti-β-Actin (1:5000; Santa Cruz Biotechnology, SC-81178). After incubation, membranes were washed 5 x 5 min with TBST, incubated in blocking buffer for 30 min, and then incubated for 90 min with either horse-radish peroxidase (HRP) conjugated goat anti-rabbit IgG (1:10000; EMD Millipore, AP307P) or HRP conjugated goat anti-mouse IgG (1:10000; Thermo Fisher Scientific, 31430). Blots were washed 5 x 5 min with TBST, and processed for chemiluminescence using SuperSignal West Dura Extended Duration Substrate (Thermo Fisher Scientific, 34075) prior to developing.

All densitometric analyses were done using Image J 1.52q software (National Institute of Health and the Laboratory for Optical and Computational Instrumentation) as previously reported [48]. Briefly, scanned Western blot images were opened in Image J and bands were selected using the rectangular selection tool. Lanes were then plotted on a histogram by selecting “Analyze-Gels-Plot Lanes.” Using the straight-line selection tool and wand tool, peaks were highlighted. Area of peaks were then measured by going to “Analyze-Gels-Label Peaks.” Data was normalized to the control band and β-Actin loading control and reported as fold increase.

### Statistical analyses

Results are presented as average ± SEM. Student’s t-test or one-way analysis of variance (ANOVA) followed by Tukey’s Multiple comparisons test were utilized for data analyses. All statistical tests were run using Prism software, version 6 (GraphPad, Inc). Results between-groups were considered statistically significant at P<0.05.

## Results

### AOAH is expressed in cortical microglia

**Fig 1:**
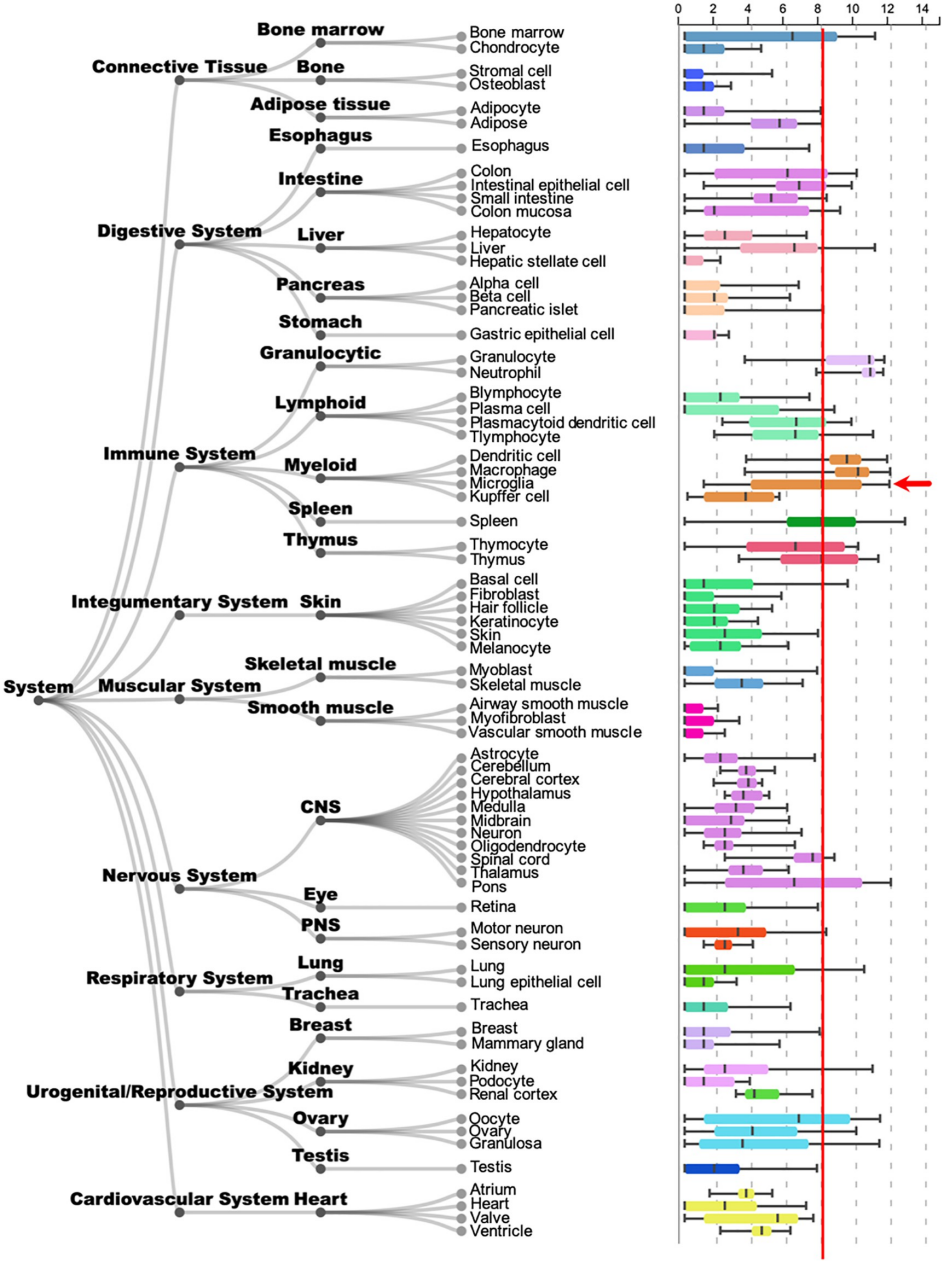
Tissue expression of *Aoah*/*AOAH* mRNA. Boxplot obtained from the public RNAseq data analysis tool ARCHS4 showing *Aoah*/*AOAH* expression in different tissue types. For comparison of microglia (arrow) relative to other cell types, approximate mean microglial expression is indicated (red line) [41].

To investigate whether AOAH potentially plays a functional role in microglia, we evaluated the possibility of *Aoah* expression in microglia using the ARCHS4 web resource of RNA-seq data. ARCHS4 is a convenient tool to identify gene expression averaged across cells and tissues from 103,083 mouse samples and 84,863 samples [41]. *Aoah*/*AOAH* was expressed in numerous tissues, including the CNS and myeloid tissue. Microglial cells showed the fifth highest level of *Aoah*/*AOAH* expression among all 72 cell and tissue types analyzed. Relative to microglia, other cells within the CNS showed modest levels of *Aoah*/*AOAH* expression, with the lowest levels observed in astrocytes (Fig 1). These data suggest that AOAH may play a role in microglial physiology.

**Fig 2:**
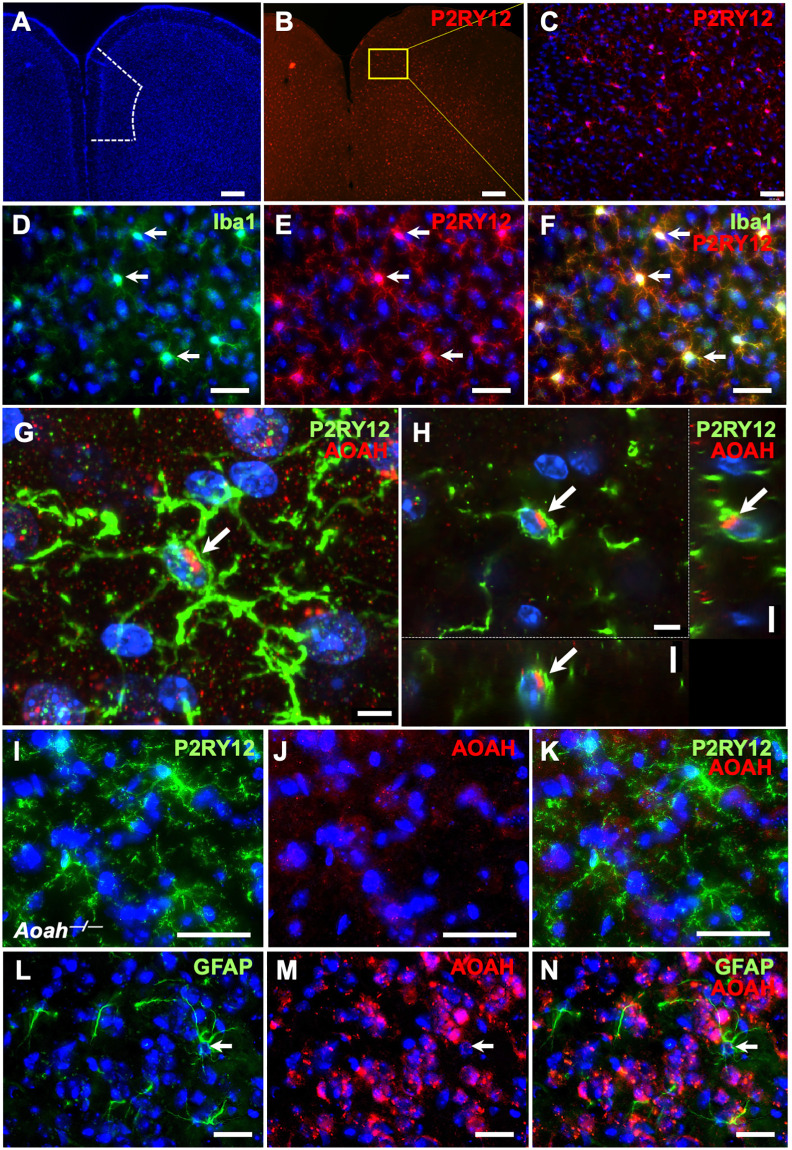
Immunostaining of AOAH in glial cells. **A-C**: P2RY12 staining labels microglia in murine P2RY12+ cells (red) in and beyond the prefrontal cortex (A, prefrontal cortex outlined in a single hemisphere by dotted line). **A** and **B** captured with 2.5X objective (scale bar: 120μm). **C** captured with a 20X objective (scale bar: 15μm). **D-F**: Combined z-stack of WT mouse prefrontal cortex stained for the microglial proteins Iba1 (green) and P2RY12 (red). DAPI staining nuclei shown in blue (n = 3 mice, scale bar: 30 μm). Images were captured with a 40X objective. **G and H**: Confocal microscopy showing combined z-stack (G) and individual planes (H) obtained from WT mouse prefrontal cortex stained for microglial protein P2RY12 (green) and AOAH (red). DAPI staining nuclei shown in blue (n = 3 mice, scale bar: 5 μm). Images were captured with a 60X objective. **I-K**: Staining of AOAH-deficient prefrontal cortex with anti-AOAH antibodies revealed an absence of AOAH (red) immunoreactivity in P2RY12+ cells (green; scale bar: 30 μm). Z-stacks were captured with a 40X objective. n = 3 mice. **L-N**: Combined z-stack of WT mouse prefrontal cortex stained for the astrocyte protein glial fibrillary acidic protein (GFAP, green) and AOAH (red). DAPI staining nuclei shown in blue (n = 3 mice, scale bar: 30 μm). Images were taken with a 40X objective.

We have previously reported AOAH protein expression in NeuN-positive neurons as well as Purkinje cells of the cerebellum [6]. To identify whether AOAH protein is also expressed in glial cells, we performed immunohistochemistry on cortical sections from wild type mice, focusing on the prefrontal cortex and visualizing microglial by staining for purinergic receptor P2Y12 (P2RY12; Fig 2A–2C). We observed complete colocalization of cell body staining with anti-P2RY12 in cortical microglia and immunostaining for ionized calcium binding adaptor molecule 1 (Iba1; Fig 2D–2F). P2RY12 is expressed exclusively on microglia in the brain, whereas Iba1 can be expressed by microglia, perivascular macrophages, and invading monocytes [49, 50]. Given this greater reported specify for microglia and our observations of brighter cell bodies and processes when immunostaining for P2RY12 (Fig 2E compared to 2D), we utilized anti-P2RY12 to identify and characterize microglia for the remainder of our experiments.

Immunohistochemistry and confocal microscopy in cortical sections revealed AOAH expression in P2RY12-positive microglia, mainly within the perinuclear region (Fig 2G for combined z-stack and 2H for individual planes), which was not observed in AOAH-deficient cortex (Fig 2I–2K). In contrast, glial fibrillary acidic protein (GFAP)-positive astrocytes showed low-to-background expression of AOAH protein (Fig 2F–2H), consistent with the relatively low *Aoah*/*AOAH* mRNA expression identified by RNA-seq in these cells as determined by ARCHS4 (Fig 1). Our initial findings indicate that AOAH may play a biological role in basal microglial homeostasis or activation.

### CNS microglia ablation reduces pelvic allodynia of AOAH-deficient mice

**Fig 3:**
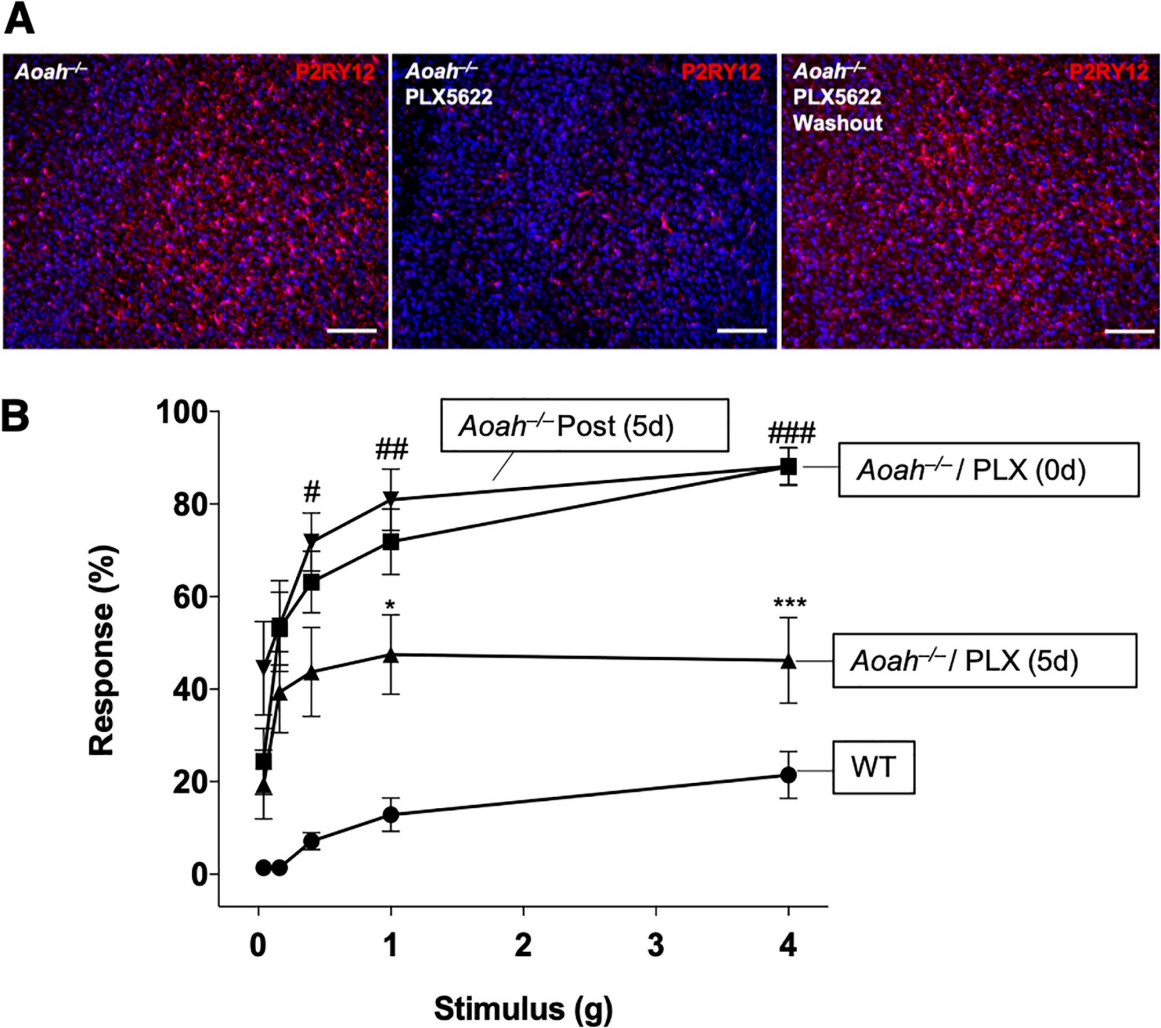
Pharmacologic ablation of microglia reduces pelvic allodynia of AOAH-deficient mice. **A**: Immunostaining of P2RY12 in AOAH-deficient prefrontal cortex from mice that were untreated (left panel), treated with 90 mg/kg of PLX5622 for 5d (middle panel) to eliminate central nervous system (CNS) microglia, or treated with PLX5622 for 5d followed by washout for 5d (right panel). DAPI staining nuclei shown in blue (scale bar: 30 μm). Z-stacks were taken with a 10X objective. **B**: Stimulating the pelvic region with von Frey filaments revealed increased response in untreated and post-treated AOAH-deficient mice compared to WT and AOAH-deficient mice that were administered 90 mg/kg of PLX5622 for 5 days by oral gavage to eliminate CNS microglia (n = 7 mice for WT, n = 16 for baseline and PLX5622-treated AOAH-deficient mice, n = 11 mice for AOAH-deficient mice post-PLX5622 treatment; *P = 0.0365, ***P = 0.0003 PLX5622 treatment compared to AOAH-deficient baseline, #P = 0.0380, ##P = 0.0089, ###P = 0.0014 Post (5 days) PLX5622 treatment compared to AOAH-deficient baseline, One-Way ANOVA followed by post-hoc Tukey HSD). Data represented as average response (%) ± SEM.

Microglia contribute significantly to the pathophysiology underlying chronic pain [20]. Our lab has identified that mice deficient for AOAH exhibit symptoms and comorbidities similarly observed in patients with IC/BPS, including pelvic pain [6]. To identify whether microglia may play a role in the pelvic pain phenotype of AOAH deficient mice, we used a pharmacological approach where antagonism of CSF1R for 5 days has been shown previously to eliminate a majority of brain microglia in mice [43]. Microglia were ablated in AOAH-deficient mice via gavage of the CSF1R inhibitor PLX5622 (90 mg/kg) for 5 days (Fig 3A, left vs. middle panel). In a pilot test of PLX5622 administration, we observed that 72% of CNS microglia were eliminated in AOAH-deficient mice after 5 days of administration (S1 Fig). Allodynia was quantified in response to von Frey filaments applied to the pelvic region using the approach of Laird and colleagues optimized to quantify allodynia on hair-covered surfaces [44]. Similar to our previous observations [6, 19], we observed that application of von Frey filaments elicited significantly more responses in AOAH-deficient mice was significantly elevated compared to wild type (Fig 3B), consistent with pelvic allodynia. AOAH-deficient mice that received PLX5622 treatment showed a 45% reduction in pelvic allodynia compared to baseline AOAH-deficient mice in response to the highest stimulus (Fig 3B, 88.13 ± 4.11% response at baseline vs 46.25 ± 9.30% response after treatment, p = 0.0003). A 34% reduction was observed after PLX5622 treatment in response to the second highest stimulus (Fig 3B, 71.88 ± 7.08% response at baseline vs 47.50 ± 8.59% response after treatment, p = 0.0365). After microglial depletion in AOAH-deficient mice, we withdrew treatment for 5 days to allow for repopulation of CNS microglia (Fig 3A, right panel and S1 Fig) as previously described in Rice et al. [51]. Microglial repopulation resulted in a significant increase in pelvic allodynia compared to treated animals in response to the three highest stimuli (71.82 ± 6.30%, 80.91 ± 6.67%, 88.18 ± 4.00% response from lowest to highest filament, p = 0.0380, 0.0089, and 0.0014 respectively), similar to our observations at baseline for AOAH-deficient mice (Fig 3B). These data suggest that microglia, in part, play a role in the pelvic pain phenotype observed in AOAH-deficient mice.

### AOAH-deficient mice exhibit activated microglial morphology

Microglia are highly dynamic cells that respond to their environment by exhibiting distinct morphological changes upon activation, where ramified microglia are considered to be in surveillance-mode and less ramified (bushy or ameboid) microglia are considered to be activated [52]. Since we observed microglial-dependent pelvic pain in AOAH-deficient mice, we next sought to identify whether AOAH-deficient microglia possessed an activated phenotype.

**Fig 4:**
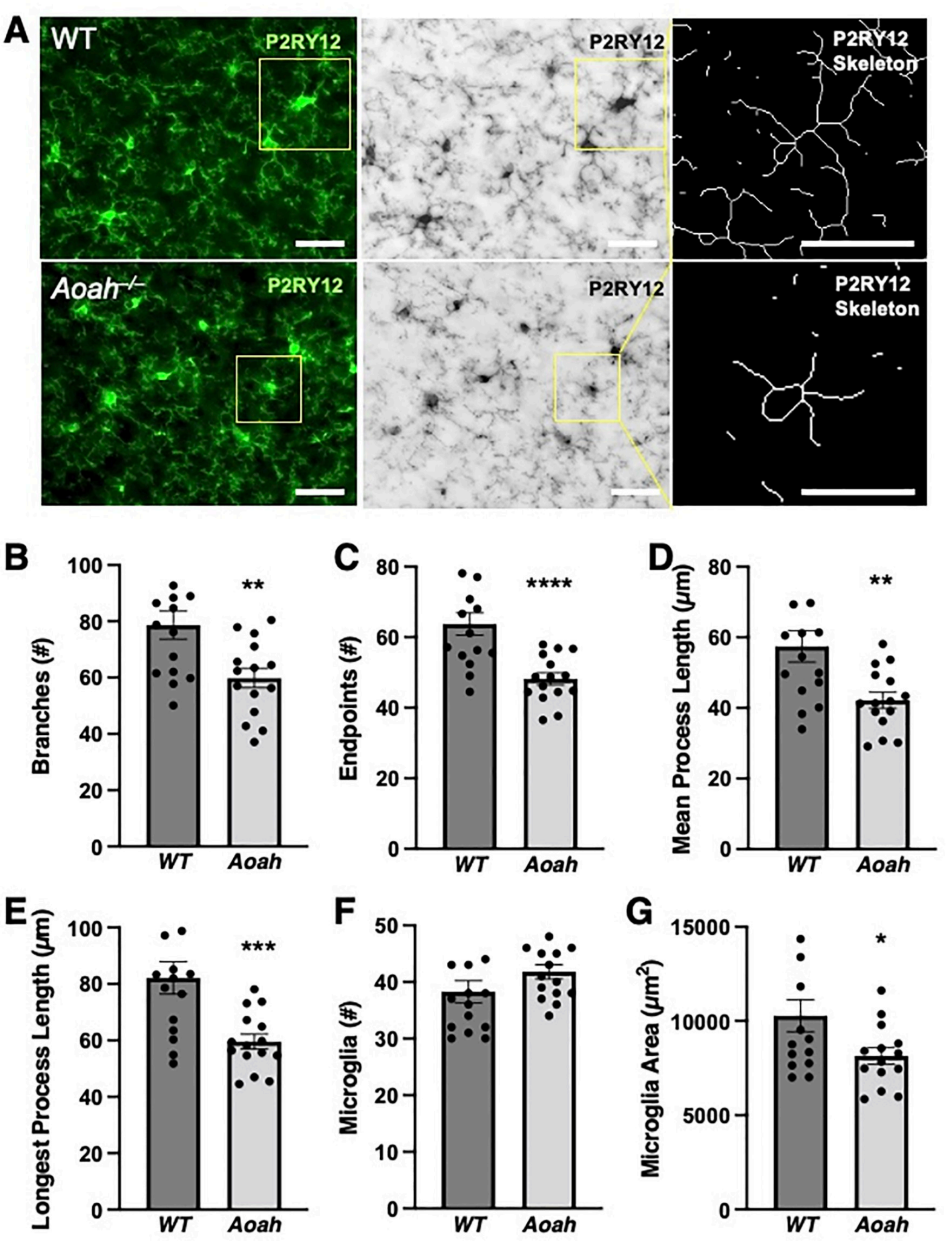
Microglia in AOAH-deficient prefrontal cortex exhibit an activated phenotype. **A**: Example of photomicrographs used for skeletal analyses obtained from the prefrontal cortex as defined in Fig 2A. Left column shows immunostaining of P2RY12 (green) in cortical microglial cells in WT (top) and AOAH-deficient (bottom) mice. DAPI staining nuclei shown in blue (scale bar: 30 μm). Middle column shows 8-bit grayscale images (scale bar: 30 μm). Right column shows example of skeletonized microglia used for quantification (scale bar: 30 μm). All z-stacks were taken with a 20X objective. **B-G**: Quantitative analyses of skeletonized microglia in the prefrontal cortex of female wild type and *Aoah*^–/–^mice (Student’s t test, two tailed. Data represented as average ± SEM; n = 15 fields from 3 mice). *Aoah*^–/–^mice exhibit significantly fewer branches (**B**, P = 0.0021), endpoints (**C**, P<0.001), mean process length (**D**, P = 0022), longest process (**E**, P = 0.0004), and microglia area (**G**, P = 0.0437). Microglia counts (**F**, P-0.192) were not significantly different.

To assess the role of AOAH on microglial morphology, brain sections were stained with the microglial marker P2RY12 followed by image processing and skeletal analyses using ImageJ (Fig 4A and S2A Fig for example of photomicrographs analyzed; all data were obtained within the prefrontal cortex as defined in Fig 2A). Skeletal analyses of microglia in the prefrontal cortex revealed that AOAH-deficient mice exhibit microglia with fewer branches (p = 0.0021, Student’s t test, two-tailed), fewer endpoints (p = <0.0001, Student’s t test, two-tailed), shorter processes (p = 0.0022 and 0.0004, Student’s t test, two-tailed), and decreased microglial area (p = 0.0437, Student’s t test, two-tailed) compared to wild type cortical microglia (Fig 4). We did not observe any changes in the number of microglia between AOAH-deficient and wild type cortical microglia (p = 0.1920, Student’s t test, two-tailed). These data suggest that AOAH-deficient mice exhibit an activated microglial morphology in the prefrontal cortex.

Since AOAH-deficient mice exhibit pelvic pain and we observed activated microglia in the prefrontal cortex (Figs 3 and 4, [6]), a brain region associated with pain modulation, we next determined if microglial activation was restricted to the prefrontal cortex or also extend to another site of pain modulation, the hypothalamic paraventricular nucleus (PVN). Similar to our observations in the prefrontal cortex, AOAH-deficient microglia in the PVN exhibited altered microglial morphology as observed by decreased number of branches (p = 0.0002, Student’s t test, two-tailed), fewer endpoints (p = 0.0295, Student’s t test, two-tailed), and decreased microglial area (p = 0.0158, Student’s t test, two-tailed) compared to wild type PVN microglia (Table 1). We did not observe differences in the length of processes (p = 0.8875 and 0.9496, Student’s t test, two-tailed) or number of microglia (p = 0.2275, Student’s t test, two-tailed) in PVN microglia between wild type and AOAH-deficient mice (Table 1). Overall, although the global nature of microglial ablation by PLX5622 precludes implicating any single region or circuit, these data show activated microglia morphology in AOAH-deficient mice in brain regions associated with neuropathic pain.

**Table 1:** Microglia skeletal analyses in paraventricular nucleus.

	WT	*Aoah* ^*—/—*^	P value
**Branches (#)**	102.80 ± 5.81	66.12 ± 3.98**	0.0002
**Endpoints (#)**	64.13 ± 3.60	52.37 ± 3.07*	0.0295
**Average Process Length (μm)**	43.27 ± 4.53	44.15 ± 3.87	0.8875
**Longest Process Length (μm)**	64.62 ± 6.68	64.10 ± 4.66	0.9496
**Microglia (#)**	28.25 ± 1.72	32.71 ± 3.23	0.2275
**Microglia Area (μm** ^**2**^ **)**	14577 ± 1631	9350 ± 744.5*	0.0158

Student’s t test, two tailed. Data represented as average ± SEM

n = 7 fields from 3 mice

Our findings reveal microglial activation in AOAH-deficient mice in brain regions at least two regions associated with pain modulation, the prefrontal cortex and the PVN (Fig 4, Table 1). To determine whether microglial activation may be a global phenomenon in AOAH-deficient CNS, we performed skeletal analyses in the CA1 region of the hippocampus and identified no differences in microglial morphology between associated with AOAH deficiency (S2 Fig). These data suggest that microglial activation in AOAH-deficient mice is region-specific and includes brain regions associated with pain modulation.

### Differential activation of BV2 cells by stool slurry

**Fig 5:**
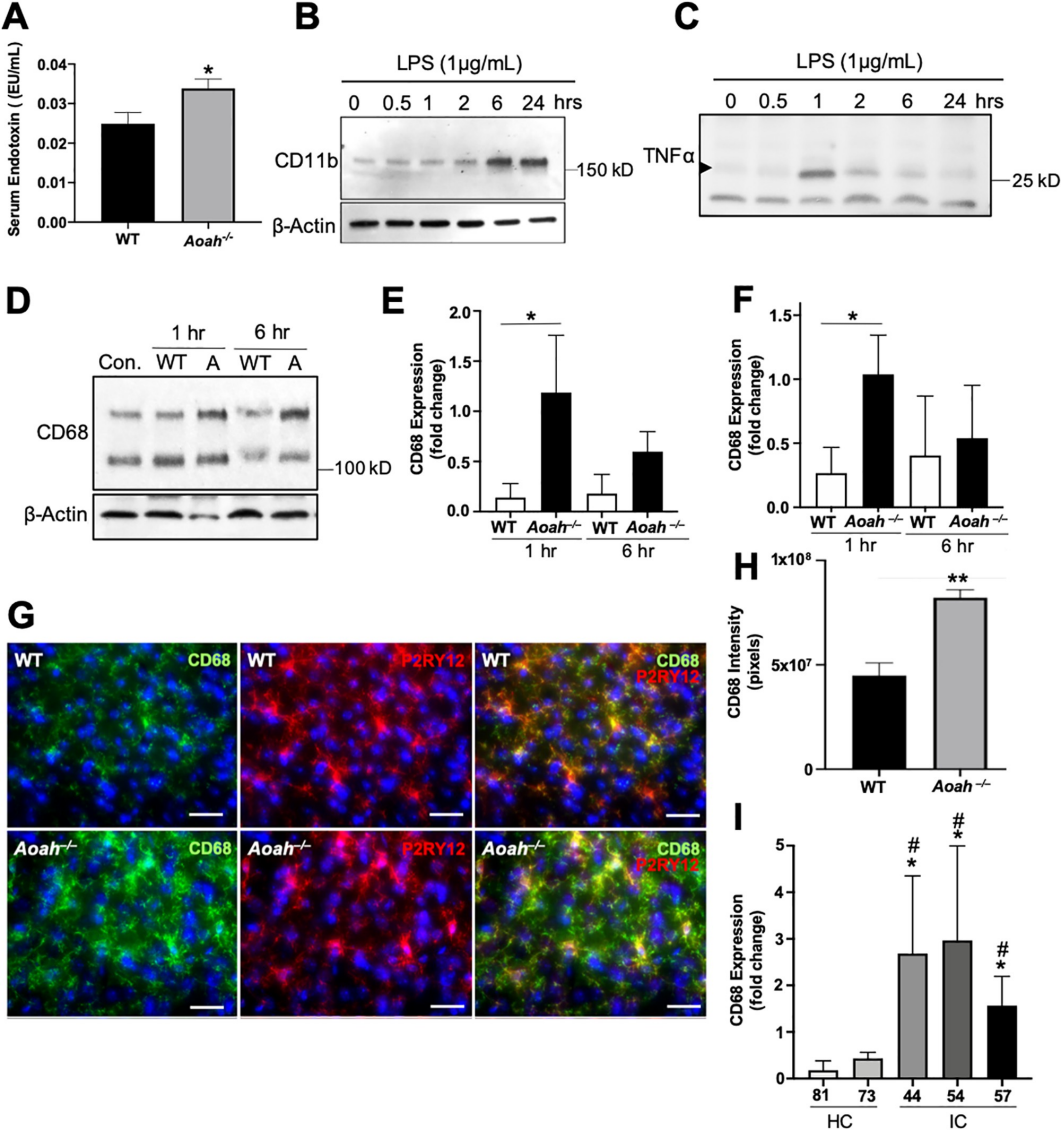
Microbiome-dependent activation of BV2 cells. **A**: Concentration of serum endotoxin in WT and AOAH-deficient mice (n = 6 mice for WT, n = 5 mice for *Aoah*^*—/—*^; *P = 0.0434, Student’s t-test, two tailed). **B**: Detergent extracts (50 μg of protein/lane) from BV2 cells activated with 1 μg/mL of LPS for 0, 0.5, 1, 2, 6, or 24 hours were analyzed by SDS-PAGE using 4–20% Tris-glycine gels followed by Western blotting. Blots were probed with CD11b (top panel, 1:2000) and β-Actin (bottom panel, 1:10000). n = 3 experiments. **C**: Cultured media (50 μg of protein/lane) from BV2 cells activated with 1μg/mL of LPS for 0, 0.5, 1, 2, 6, or 24 hours were analyzed by SDS-PAGE using 4–20% Tris-glycine gels followed by Western blotting. Blots were probed with TNFα (1:2000). n = 3 experiments. **D**: Detergent extracts (50 μg of protein/lane) from BV2 cells activated with 1 mg/mL of heat-killed stool slurry from WT (Con. and WT) or AOAH-deficient (A) stool for 0, 1, or 6 hours were analyzed by SDS-PAGE using 4–20% Tris-glycine gels followed by Western blotting. Blots were probed with CD68 (top panel, 1:2000) and β-Actin (bottom panel, 1:10000). n = 4 experiments. **E and F**: Band intensities were quantified by densitometric analysis and reported as relative levels from control baseline of the top (E) and bottom (F) bands of CD68 normalized to β-Actin (n = 4 experiments; *P = 0.05, Student’s t-test, two tailed). **G**: Immunostaining of CD68 in cortical microglial cells. Mouse prefrontal cortex from WT (top row) and AOAH-deficient (bottom row) mice were stained for the inflammatory marker CD68 (green) and the microglial marker P2RY12 (red). DAPI staining nuclei shown in blue (scale bar: 30 μm). Z-stacks were taken with a 40X objective. n = 3 mice. **H**: Quantification of CD68 immunostaining in microglia from prefrontal cortex (n = 5 for both conditions; **P = 0.0023, Student’s t-test, two tailed). Data represented as mean integrated density in pixels ± SEM. **I**: Detergent extracts (50 μg of protein/lane) from BV2 cells activated with 1 μg/mL of heat-killed stool slurry from healthy (HC) or IC/BPS (IC) patients for 1 hr were analyzed by SDS-PAGE using 4–20% Tris-glycine gels followed by Western blotting. Blots were probed with CD68 (1:2000) and β-Actin (1:10000). Band intensities were quantified by densitometric analysis and reported as relative levels from baseline of the bottom band of CD68 normalized to β-Actin (n = 3 experiments; *P<0.05 compared to 81 HC, #P<0.05 compared to 73 HC, One-Way ANOVA followed by post-hoc Tukey HSD).

We have previously shown gut dysbiosis and an impaired gut epithelial barrier in AOAH-deficient mice [19], allowing the possibility for circulating microbial constituents (such as LPS) to activate microglia. To test whether AOAH-deficient mice exhibit increased concentration of circulating endotoxins, we performed a LAL endotoxin assay on serum collected from wild type and AOAH-deficient mice. We observed a 36% increase in endotoxin concentration in AOAH-deficient serum compared to wild type (Fig 5A, p = 0.0434, Student’s t test, two-tailed), suggesting that the AOAH-deficient “leaky gut” may be releasing gut components to the bloodstream.

Since we observed increased serum endotoxin in AOAH-deficient mice (Fig 5A), we next sought to quantify differences in activation in a microglial cell line in response to gut bacteria. Stimulating BV2 cultures with LPS from *E*. *coli* resulted in increased accumulation of activation marker CD11b in cell lysates, peaking at 6 hrs of activation, and secretion of the pro-inflammatory cytokine TNF⍺ in cell medium peaking at 1 hr of activation (Fig 5B and 5C respectively). These data show that BV2 cells are activated by LPS in a time-dependent manner consistent with microglial activation.

We next determined whether BV2 cells respond differentially in response to bacteria from wild type and AOAH-deficient gut by stimulating cultures with stool slurry. Upon stimulating BV2 cultures with heat-killed stool slurry from wild type mice, we observed cell activation peaking at 1 hr as quantified by activation marker CD68 expression (Fig 5D–5F). In comparison, when stimulating with slurry from AOAH-deficient mice, cultures showed significantly greater activation at 1 hr and activation was sustained with high levels of CD68 after 6 hrs of activation, although this time point did not approach significance (Fig 5D–5F). Since CD68 glycosylation correlates with macrophage activation and leads to differential observed molecular mass [53], we quantified both lower and higher molecular mass CD68 bands and observed higher levels of both forms after activation with AOAH-deficient stool slurry at 1 hour (Fig 5E and 5F).

The increase in BV2 CD68 expression (Fig 5D–5F), a suggested marker of phagocytosis [54] implicates AOAH-deficiency in altered CD68 expression. We performed immunohistochemistry to test whether microglial CD68 is upregulated in AOAH-deficient prefrontal cortex (Fig 5G). We observed low levels of CD68 expression in microglia in wild type prefrontal cortex (Fig 5G, top row and Fig 5H). AOAH deficiency resulted in increased microglia CD68, which was observed in both the cell body and processes of microglial cells (Fig 5G, bottom row and Fig 5H). These findings suggest a role for AOAH deficiency in CD68 expression and phagocytosis.

IC/BPS patients exhibit gut dysbiosis [2]. Since CD68 is upregulated in AOAH-deficient microglia of the prefrontal cortex and by dysbiotic AOAH-deficient stool slurry activation of BV2 cells (Fig 5D–5H), we next examined whether stool slurry from IC/BPS patients could activate BV2 cells. BV2 cultures were stimulated with heat-killed slurry of stool from healthy controls or IC/BPS patients followed by immunoblotting for CD68. Greater BV2 activation was observed in cultures stimulated by stool slurry from IC/BPS patients compared to controls (Fig 5I), raising the possibility of dysbiosis-mediated microglial activation as a contributing factor to pelvic pain in IC/BPS patients. Overall, our findings show that altered stool composition and gut microbiota can differentially activate microglial cells.

## Discussion

**Fig 6:**
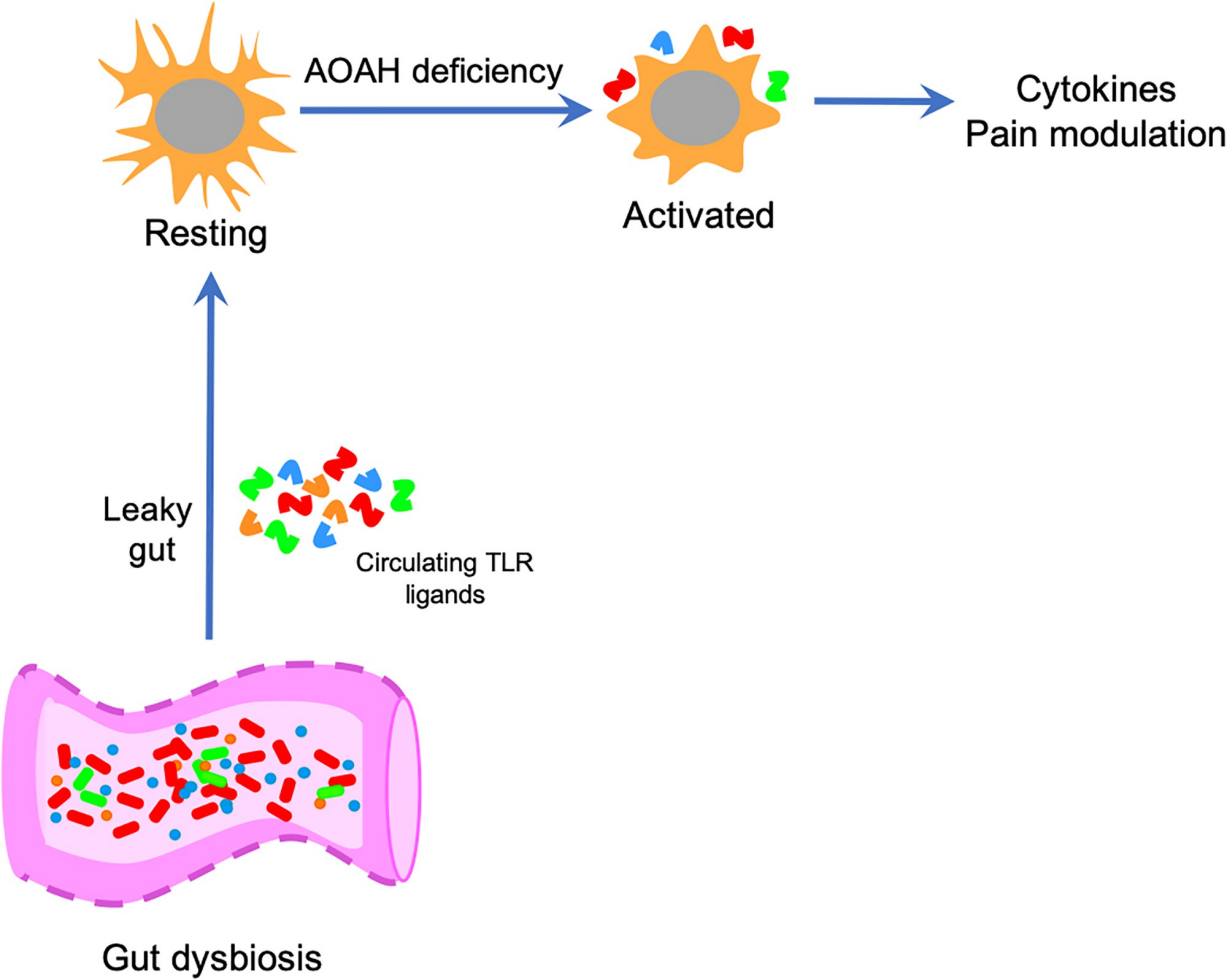
Role for AOAH in microglial activation. At baseline, microglia exhibit a ramified/resting phenotype. AOAH deficiency results in microglia with an activated phenotype. Microglial activation associated with AOAH deficiency could arise in direct response to circulating microbial ligands that the gut crossing the blood brain barrier and activating microglia, or through indirect mechanisms including enhanced CRF signaling, vagus nerve signaling to the CNS, or intrinsic hyper-responsiveness due to loss of AOAH-mediated arachidonic acid homeostasis. Prolonged microglial activation then modulates pelvic nociception.

We previously reported that AOAH-deficiency mimics several aspects of IC/BPS, including pelvic pain and gut dysbiosis [6, 15, 19]. Here, we report morphologic changes in AOAH-deficient microglia consistent with microglial activation in brain centers known to modulate nociception and reduced pelvic allodynia following pharmacologic ablation of microglia with PLX5622, although we cannot implicate specific brain regions due to the global nature of pharmacologic microglial ablation. Nonetheless, consistent with our previous findings of gut dysbiosis and decreased gut barrier function [19], here we also report elevated serum endotoxin in AOAH-deficient mice and enhanced activation of cultured microglia by AOAH-deficient microbiota. Together, these findings suggest a working model for the role of AOAH in modulating pelvic pain at the level of microglial activation in response to gut dysbiosis (Fig 6). Consistent with prior studies implicating both microglial function and dysbiosis of gut flora in mediating chronic pain [20, 33–36], we propose that microglia are transducers of dysbiosis that modulate pelvic pain. Microglia are known to express Toll-like receptors (TLRs) and thus may act as sentinels that undergo activation in response to TLR ligands [55–57]. Supporting this model, we find elevated serum endotoxin in AOAH-deficient mice (Fig 5). Targeting TLR expression in microglia in future studies will determine whether AOAH indeed modulates pelvic pain through such a transducer role.

AOAH deficiency results in microglial activation, and AOAH is poised to potentially influence microglial activation directly and/or indirectly. First, we find AOAH is expressed in microglia (Figs 1 and 2) and thus may influence intrinsic microglia physiology. For example, microglial activation has been associated with altered arachidonic acid-containing phosphatidyl choline metabolism in a neuropathic pain model [58]. We recently showed that AOAH mediates CNS arachidonic acid homeostasis at the level of sequestration in phospholipids, leading to elevated PGE2 signaling that contributes to pelvic pain [59]. This suggests that AOAH is an intrinsic mediator of microglial homeostasis, possibly at the level of arachidonic acid sequestration. In this case, AOAH deficiency may result in dysregulated arachidonic acid metabolism in microglia, potentially then shifting equilibrium toward an activated morphology observed in the cortex and PVN (Fig 4 and Table 1). Alternatively, we have previously identified *Aoah* as a genetic regulator of corticotropin releasing factor (CRF) in the PVN [12], a pertinent brain region for CRF-dependent pain modulation and stress responses [13, 14]. AOAH-deficient mice exhibit increased neuronal *Crf* expression sufficient to drive elevated corticosterone levels, indicative of hypothalamic-pituitary-adrenal axis disruption, and resulting in an anxious/depressive phenotype [12]. Microglia are themselves responsive to CRF, where CRF receptor engagement triggers microglial proliferation and cytokine release [60, 61]. Together, these findings suggest that the increased neuronal CRF release in AOAH-deficient mice may contribute to microglial activation. Consistent with this possibility, neuronal CRF release and microglial activation in the PVN have been observed in a rodent model of irritable bowel syndrome [62]. Any of these mechanisms could be exacerbated by the possibility of elevated serum endotoxin crossing the blood-brain barrier and thereby contributing to TLR-mediated microglial activation. Thus, by direct and/or indirect mechanisms, AOAH deficiency contributes to microglial activation, acting in concert with nociceptive inputs such as bladder mast cell activation and elevated PGE2 signaling [6, 59] to mediate pelvic pain. Future studies targeting AOAH expression in microglia will inform these mechanisms.

These studies and prior reports paint an emerging picture of microglia involvement in pelvic pain. For example, chronic prostatitis/chronic pelvic pain syndrome (CP/CPPS) is a urologic chronic pelvic pain syndrome (UCPPS) analogous to IC/BPS that is also associated with severe pelvic pain and anxiety/depression [63]. Experimental autoimmune prostatitis (EAP) is a clinically relevant model of CP/CPPS where microglia activation has been observed in the spinal cord and brain [28–30] thus raising the possibility of microglial modulation of pelvic pain along the genitourinary-brain axis constituting the nociceptive pathway. For example, microglial activation was identified in spinal cord of EAP mice and associated with increased brain-derived neurotrophic factor (BDNF) expression, and spinal dorsal horn microglia were similarly activated in mice treated with cyclophosphamide to induce cystitis [30, 64]. Similarly, brains of EAP mice revealed overall increased Iba1 staining across the hippocampus, consistent with microglial activation, and associated pelvic allodynia and cognitive deficits [28]. This is in slight contrast to AOAH-deficient mice that exhibit no evidence for microglial activation of CA1 microglia (S2 Fig) but instead show hippocampal microglia exhibiting morphologic markers of activation limited to the dentate gyrus (Rahman-Enyart, in preparation). While these differences may reflect sex differences or nuances of model systems or modes of analyses, together these findings are consistent with evidence for microglial activation across a range of clinically relevant UCPPS models mimicking IC/BPS and CP/CPPS. Indeed, we find microglial activation in the PVN and prefrontal cortex of AOAH-deficient mice (Fig 4 and Table 1). In rodents, the PVN modulates voiding and bladder nociception through connectivity with the Barrington’s nucleus and the periaquaductal gray (PAG, [65]). Since PAG is also a site of increased activity in UCPPS patients, this raises the intriguing possibility that PVN microglia may indirectly modulate PAG activity in UCPPS patients. Likewise, we note activation of prefrontal cortex microglia, and CP/CPPS is associated with reduced gray matter volume in the anatomically related anterior cingulate cortex, potentially evidence of synaptic remodeling, an activity of microglia [66–68]. Thus, there is increasing evidence for microglial modulation of pelvic pain and potential implications. However, microglia-neuron interactions should be further explored to identify the potential effects of microglial activation on neuronal circuits in these model systems of pelvic pain.

AOAH-deficient mice exhibit gut dysbiosis, including an enrichment in gut bacteria, where microbiota manipulation can alleviate pelvic allodynia and anxious behavior [19]. AOAH deficiency is also associated with decreased trans-epithelial electrical resistance, and we find elevated serum endotoxin, together suggesting a leaky gut phenotype in AOAH-deficient mice (Fig 5 and [19]). In addition, here we observed that exposure to AOAH-deficient stool slurry resulted in increased activation of BV2 microglial cells *in vitro* relative to wild type stool, and that microglial ablation improved pelvic pain (Figs 2 and 5). These findings suggest a role for gut microbe-microglia interactions in pelvic allodynia, which may be regulated by gut composition. The gut microbiome of AOAH-deficient mice exhibits significantly increased abundance of several bacterial phyla compared to wild-type mice, including the presence of cyanobacteria [19]. A previous study by Mayer and colleagues exposed rat microglia *in vitro* with cyanobacterium *Oscillatoria* sp. LPS and observed a concentration-dependent release of both pro- and anti-inflammatory cytokines and chemokines [69]. Therefore, the interaction between strains of cyanobacteria and microglia may contribute to the pelvic pain phenotype of AOAH-deficient mice. Because IC/BPS patients also exhibit gut dysbiosis, we speculate similar mechanisms of dysbiosis-associated microglia activation also contributing to pelvic pain in UCPPS.

Microglial activation has been observed in various clinical disorders including incidence of anxiety and depression [70–72], disorders that are comorbid with IC/BPS [16–18]. In addition, AOAH-deficient mice exhibit neophobia and anhedonia-like behaviors, both of which are associated with anxiety and depression [12], thus drawing additional parallels with IC/BPS. Human studies have suggested microglial activation may play an important role in depression. For example, postmortem brain analyses in depressed patients and suicide victims reveal increased microgliosis and a higher ratio of primed over ramified (resting) microglia [73–75]. Microgliosis has been observed in brains of suicide victims in several regions, such as the dorsolateral prefrontal cortex, anterior cingulate cortex, and mediodorsal thalamus [73, 74]. Whether microglial activation is associated with IC/BPS is currently unknown, but the parallels between AOAH-deficient mice and IC/BPS raise the possibility that microglia activation may play a role in pelvic pain for at least a subset of these patients. Indeed, gut dysbiosis in AOAH-deficient mice is associated with increased gut permeability that is consistent with the elevated serum endotoxin reported here and the possibility of microglial activation via consequent leakage across the blood-brain barrier. In addition, we have demonstrated that stimulating microglial BV2 cells with stool slurry from IC/BPS patients resulted in upregulation of the microglial activation marker CD68 (Fig 5). These *in vitro* findings suggest a potential role for gut microbiome-microglia interactions in IC/BPS, possibly regulating the pelvic pain phenotype by elevated serum endotoxin crossing the blood-brain barrier in patients. As a result, therapies that regulate microglial homeostasis or target microglial effectors of pain modulation (e.g., cytokines) offer promise for treating IC/BPS.

In summary, the data presented here show that AOAH is a mediator of microglia homeostasis in brain regions known to modulate pain. These findings demonstrate microglial regulators as promising pharmacological targets for treating pelvic pain.

## Supporting information

S1 Fig Quantification of microglial depletion in response to PLX5622 administration. **A-I**: Immunostaining of P2RY12 in AOAH-deficient prefrontal cortex (Cx, A, D, and G), PVN (B, E, and H) and the CA1 region of the hippocampus (Hi, C, F, and I) from mice that were untreated (left column), treated with 90 mg/kg of PLX5622 for 5d (middle column) to eliminate central nervous system (CNS) microglia, or treated with PLX5622 for 5d followed by washout for 5d (right column). DAPI staining nuclei shown in blue (scale bar: 30 μm). Z-stacks were taken with a 40X objective. **J**: Percentage of total microglia in combined z-stacks from three AOAH-deficient brain regions (prefrontal cortex, PVN, and the CA1 region of the hippocampus) from mice that were untreated (control), treated with 90 mg/kg of PLX5622 for 5d (PLX5622), or treated with PLX5622 for 5d followed by washout for 5d (washout). n = 1 mouse/condition.
(TIFF)

S2 Fig Microglia are not activated in CA1 region of the hippocampus in AOAH-deficient mice. **A**: Example of photomicrographs used for skeletal analyses. Left column shows immunostaining of P2RY12 (green) in cortical microglial cells in WT (top) and AOAH-deficient (bottom) mice. DAPI staining nuclei shown in blue (scale bar: 30 μm). Middle column shows 8-bit grayscale images (scale bar: 30 μm). Right column shows example of skeletonized microglia used for quantification (scale bar: 30 μm). All z-stacks were taken with a 20X objective. **B-F**: Skeletal analyses in the CA1 region of the hippocampus in WT and AOAH-deficient mice revealed no changes in the number of branches (B), number of endpoints (C), average process length (D), longest process length (E), and number of microglia (F) between conditions (n = 11 fields from 3 mice; P>0.05, Student’s t-test, two tailed).
(TIFF)

S3 Fig Raw images of immunoblots. Photos show all bands in context of developed films and regions cropped for display in Fig 5B–5D.
(PDF)

S1 File Methods for quantification of microglial depletion, and demographic information and GUPI scores of healthy controls and IC patients. (DOCX)

S2 File Raw morphometry data for microglia of the prefrontal cortex. (XLSX)

S3 File Raw morphometry data for microglia of the paraventricular nucleus. (XLSX)
