# Supplementary figures and images for: Acyloxyacyl hydrolase regulates microglia-mediated pelvic pain
Source: PLoS One. 2022 Aug 18;17(8):e0269140. doi: 10.1371/journal.pone.0269140 (PMC9387837; doi:10.1371/journal.pone.0269140)

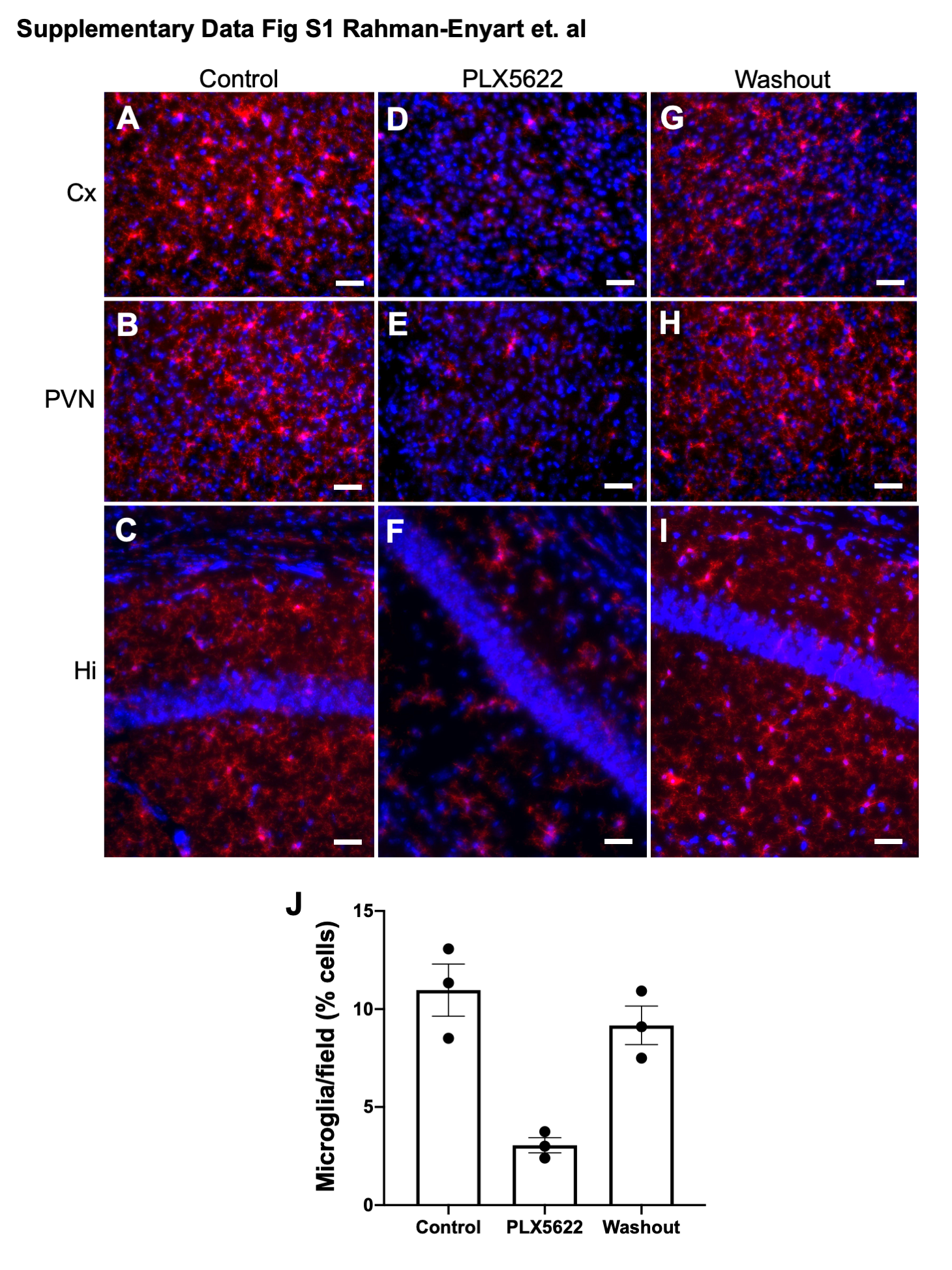

Supplement: S1 Fig — A-I: Immunostaining of P2RY12 in AOAH-deficient prefrontal cortex (Cx, A, D, and G), PVN (B, E, and H) and the CA1 region of the hippocampus (Hi, C, F, and I) from mice that were untreated (left column), treated with 90 mg/kg of PLX5622 for 5d (middle column) to eliminate central nervous system (CNS) microglia, or treated with PLX5622 for 5d followed by washout for 5d (right column). DAPI staining nuclei shown in blue (scale bar: 30 μm). Z-stacks were taken with a 40X objective. J: Percentage of total microglia in combined z-stacks from three AOAH-deficient brain regions (prefrontal cortex, PVN, and the CA1 region of the hippocampus) from mice that were untreated (control), treated with 90 mg/kg of PLX5622 for 5d (PLX5622), or treated with PLX5622 for 5d followed by washout for 5d (washout). n = 1 mouse/condition. (TIFF) [file pone.0269140.s001.tiff]

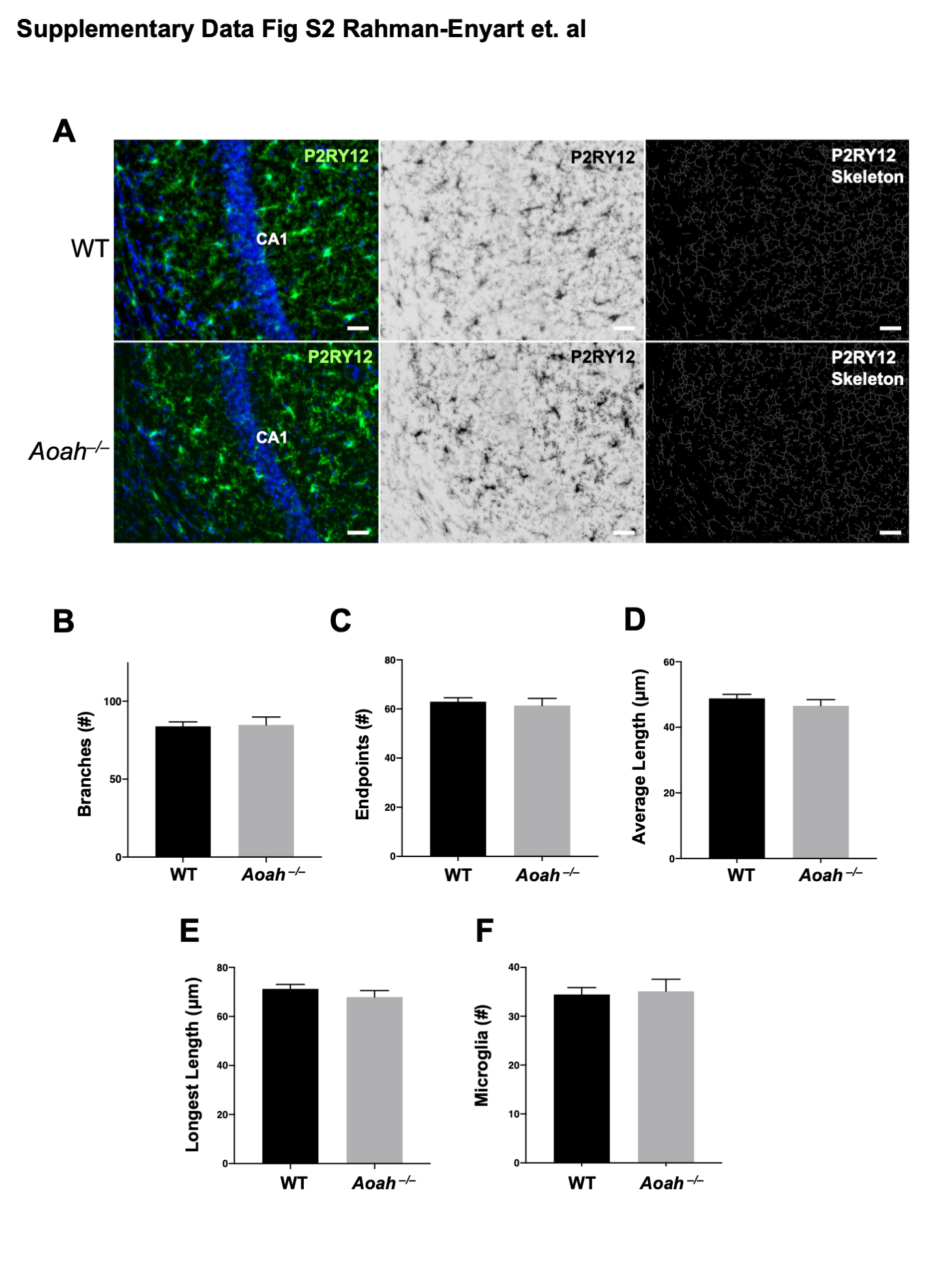

Supplement: S2 Fig — A: Example of photomicrographs used for skeletal analyses. Left column shows immunostaining of P2RY12 (green) in cortical microglial cells in WT (top) and AOAH-deficient (bottom) mice. DAPI staining nuclei shown in blue (scale bar: 30 μm). Middle column shows 8-bit grayscale images (scale bar: 30 μm). Right column shows example of skeletonized microglia used for quantification (scale bar: 30 μm). All z-stacks were taken with a 20X objective. B-F: Skeletal analyses in the CA1 region of the hippocampus in WT and AOAH-deficient mice revealed no changes in the number of branches (B), number of endpoints (C), average process length (D), longest process length (E), and number of microglia (F) between conditions (n = 11 fields from 3 mice; P>0.05, Student’s t-test, two tailed). (TIFF) [file pone.0269140.s002.tiff]
