# Supplementary material for: Acyloxyacyl hydrolase regulates microglia-mediated pelvic pain
Source: PLoS One. 2022 Aug 18;17(8):e0269140. doi: 10.1371/journal.pone.0269140 (PMC9387837; doi:10.1371/journal.pone.0269140)

Full, unedited blot
for Fig 5B

CD11b blot

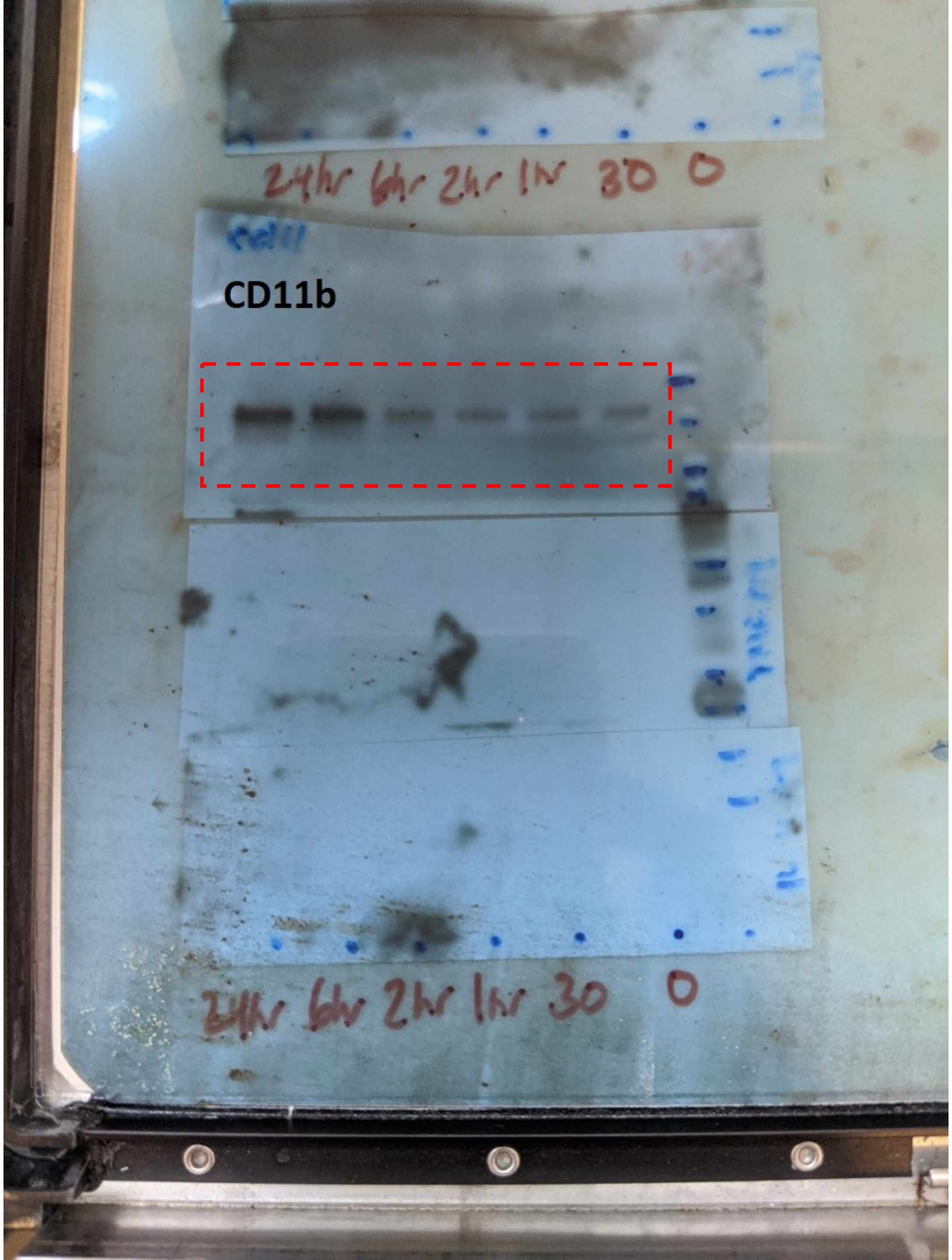

Full, unedited blot
for Fig 5B

Actin re-probe of
CD11b blot

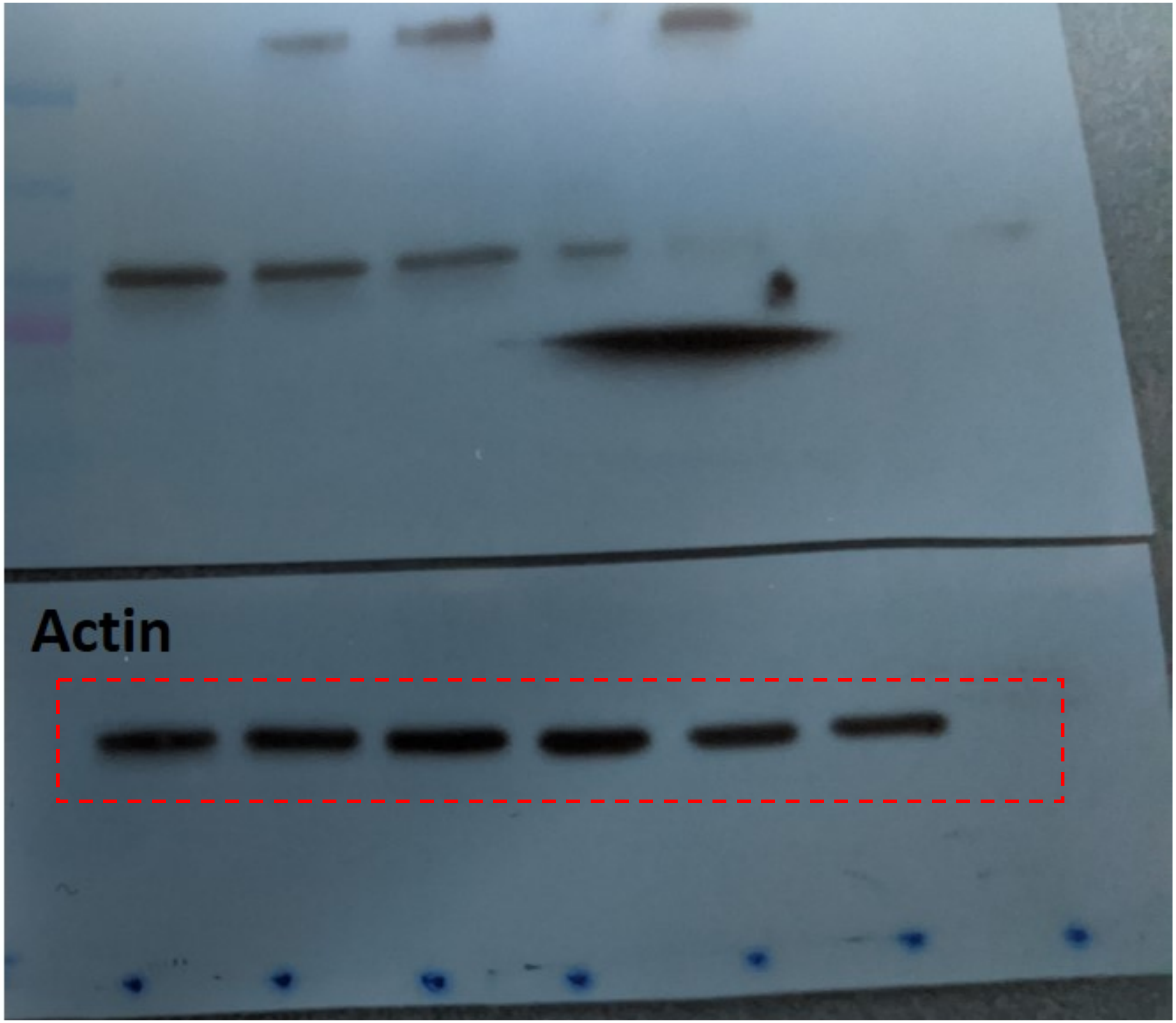

Full, unedited blot
for Fig 5C

TNF blot

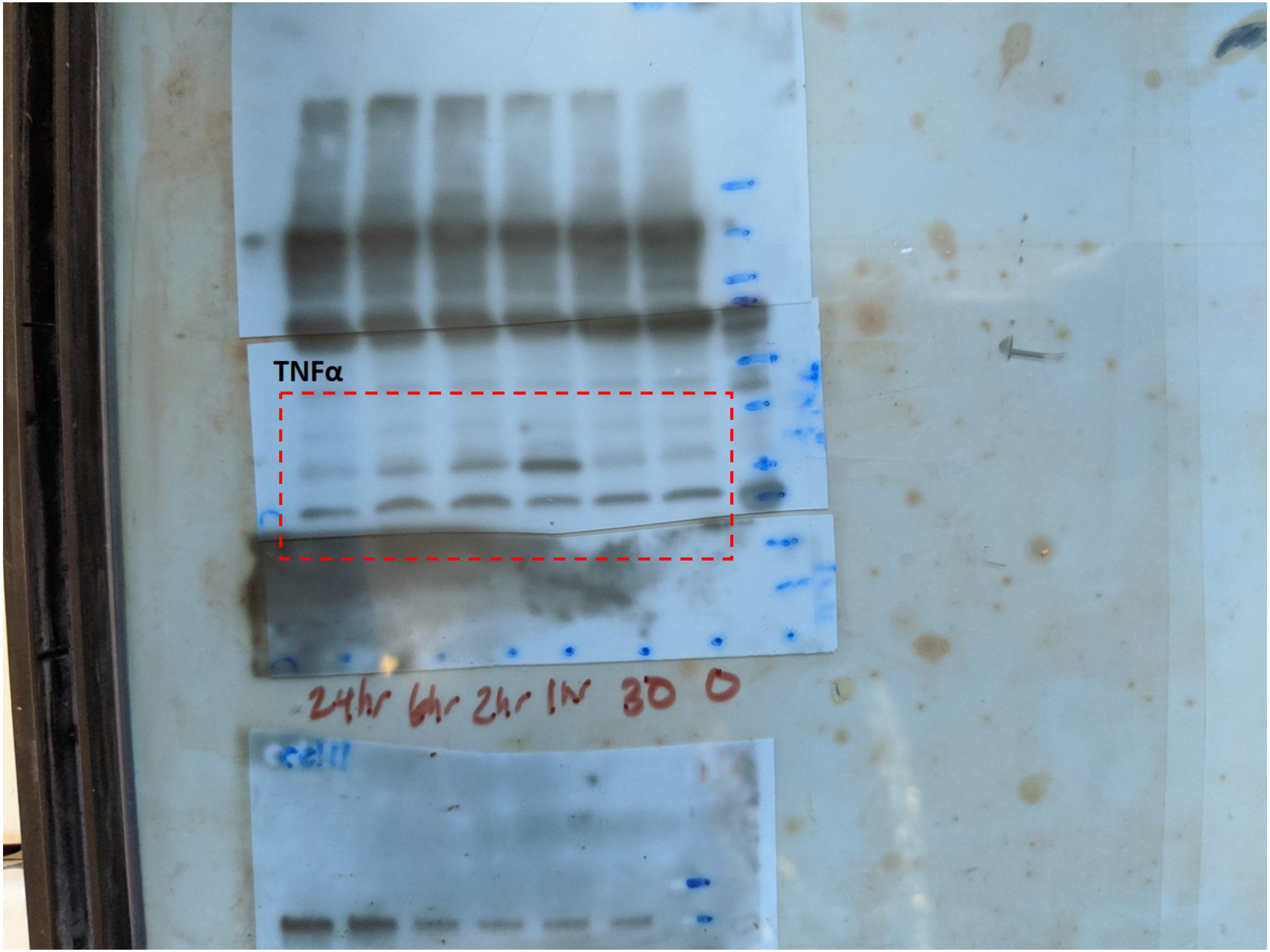

Full, unedited blot
for Fig 5D

CD68 blot

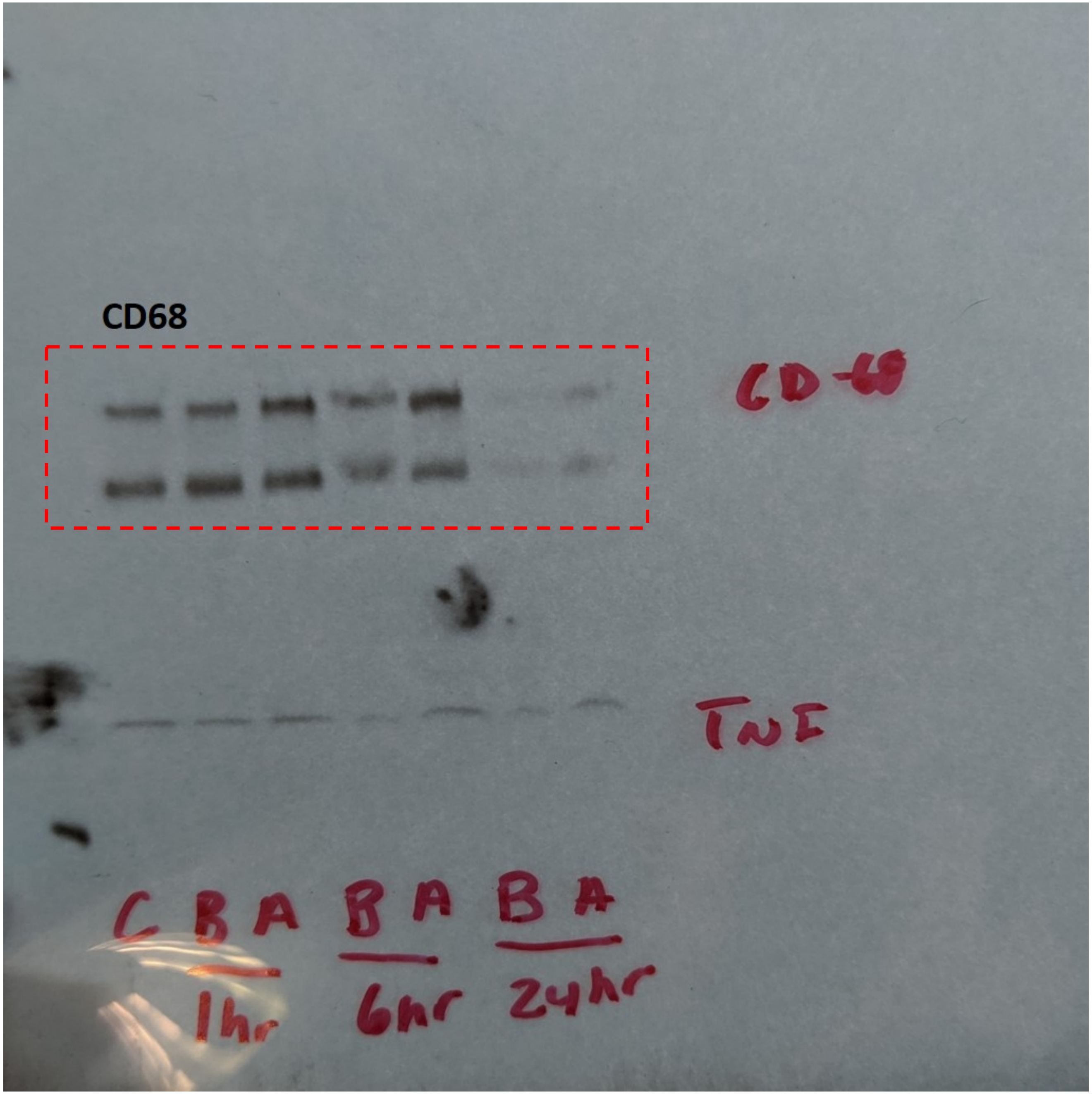

Full, unedited blot
for Fig 5D

Actin re-probe of
CD68 blot

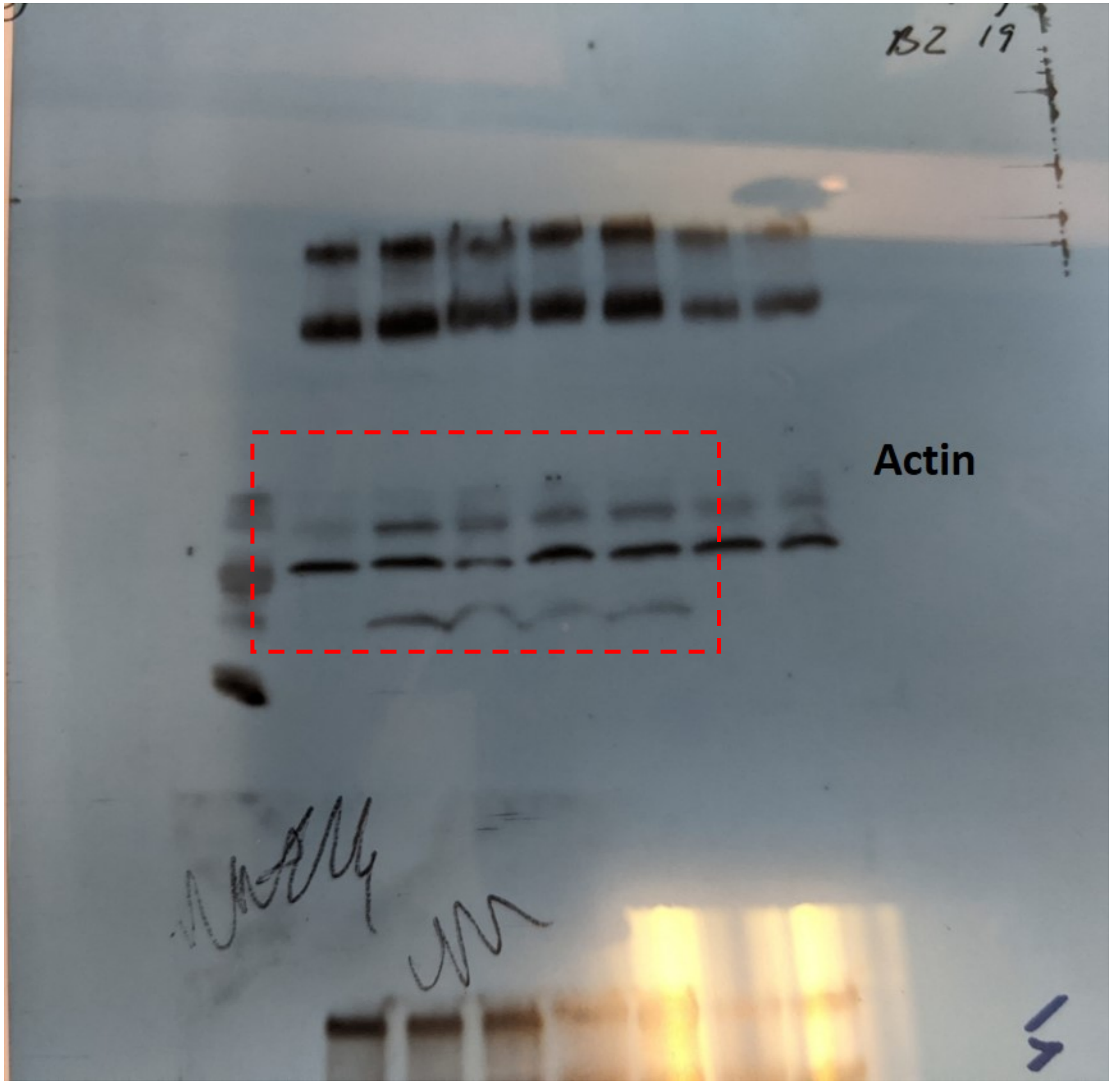

Supplement: S3 Fig — Photos show all bands in context of developed films and regions cropped for display in Fig 5B–5D. (PDF) [file pone.0269140.s003.pdf]
