# Supplementary material for: Acyloxyacyl hydrolase regulates microglia-mediated pelvic pain
Source: PLoS One. 2022 Aug 18;17(8):e0269140. doi: 10.1371/journal.pone.0269140 (PMC9387837; doi:10.1371/journal.pone.0269140)
Supplement: S1 File — (DOCX) [file pone.0269140.s004.docx]

**SUPPLEMENTARY METHODS**

**Quantification of microglial depletion.** Total number of P2RY12+ microglia and total number of DAPI+ cells were counted in a pilot test of PLX5622 administration (See manuscript sections “Immunohistochemistry” and “PLX5622 treatment” for details on staining and drug administration, respectively). Three different brain regions (the prefrontal cortex, the PVN, and the CA1 region of the hippocampus) were analyzed in AOAH-deficient mice that were untreated (n=1), treated with 90 mg/kg of PLX5622 for 5d (n=1), or treated with PLX5622 for 5d followed by washout for 5d (n=1). Cells were counted by opening combined z-stacks in Image J and utilizing the Image J Cell Counter tool. Percentage of microglia were calculated using the following equation:

**%microglia per field** = (# of microglia/# of all cells)*100

Data is reported as average microglia/field (%) ± SEM in all three brain regions analyzed.

**Table S1. Patient Information**

|  | Healthy Control | | IC Patients | | |
| --- | --- | --- | --- | --- | --- |
| Patient # | 81 | 73 | 44 | 54 | 57 |
| Age (yrs) | 24 | 37 | 50 | 30 | 50 |
| Sex | F | F | F | F | F |
| Race | Latino, Caucasian | African American | African American | Caucasian | Latino, Caucasian |
| Genitourinary urinary pain index (GUPI), total | 0 | 0 | 32 | 31 | 31 |
| GUPI, pain subscore | 0 | 0 | 14 | 14 | 17 |
| GUPI, urinary symptoms subscore | 0 | 0 | 9 | 10 | 2 |
| GUPI, QoL subscore | 0 | 0 | 9 | 7 | 12 |
